# Effects of Atomization Injection on Nanoparticle Processing in Suspension Plasma Spray

**DOI:** 10.3390/nano6050094

**Published:** 2016-05-20

**Authors:** Hong-bing Xiong, Cheng-yu Zhang, Kai Zhang, Xue-ming Shao

**Affiliations:** 1 Key Laboratory of Soft Machines and Smart Devices of Zhejiang Province, Zhejiang University, Hangzhou 310027, China; zcy330106@outlook.com (C.Z.); zhangk_0079@163.com (K.Z.); 2 Research Institute of Petroleum Engineering, Shengli Oilfield, Sinopec, Dongying 257000, China

**Keywords:** suspension plasma spray, atomization, nanoparticles, multiphase flow, thermal spray

## Abstract

Liquid atomization is applied in nanostructure dense coating technology to inject suspended nano-size powder materials into a suspension plasma spray (SPS) torch. This paper presents the effects of the atomization parameters on the nanoparticle processing. A numerical model was developed to simulate the dynamic behaviors of the suspension droplets, the solid nanoparticles or agglomerates, as well as the interactions between them and the plasma gas. The plasma gas was calculated as compressible, multi-component, turbulent jet flow in Eulerian scheme. The droplets and the solid particles were calculated as discrete Lagrangian entities, being tracked through the spray process. The motion and thermal histories of the particles were given in this paper and their release and melting status were observed. The key parameters of atomization, including droplet size, injection angle and velocity were also analyzed. The study revealed that the nanoparticle processing in SPS preferred small droplets with better atomization and less aggregation from suspension preparation. The injection angle and velocity influenced the nanoparticle release percentage. Small angle and low initial velocity might have more nanoparticles released. Besides, the melting percentage of nanoparticles and agglomerates were studied, and the critical droplet diameter to ensure solid melting was drawn. Results showed that most released nanoparticles were well melted, but the agglomerates might be totally melted, partially melted, or even not melted at all, mainly depending on the agglomerate size. For better coating quality, the suspension droplet size should be limited to a critical droplet diameter, which was inversely proportional to the cubic root of weight content, for given critical agglomerate diameter of being totally melted.

## 1. Introduction

The technology of suspension plasma spray (SPS) is a novel spray technology [1]. In the SPS process, a liquid feedstock is used to inject nanometer-sized particles with the aid of a suspension. The suspension mixes the solid nanoparticles and the solution of water or alcohol, where the nanoparticles are usually agglomerated due to their high surface activity. Liquid feedstock spraying in general could offer unique opportunities in designing and fabricating complex material architectures with controlled and hierarchical microstructures. For example, the thermoelectric modules and solar cells were recently made from thermal sprayed silicon wafers [2]. Further, liquid feedstock spraying could lead to advancement of the spraying industry to spray nanoparticles in order to obtain dense and thick coating with good bond strength.

Suspension plasma spray process involves a series of complex phenomena, such as feedstock injection, suspension breakup and evaporation, and nanoparticles or their agglomerates release into the plasma jet [3]. The solid particles will experience further heating, melting, and evaporation before impacting onto the substrate. Researchers are attempting to analyze the above-mentioned complex phenomena in order to suggest the link between them. Modeling and numerical methods are employed to better understand the flow physics related to suspension atomization and spray evolution.

Though many experimental investigations have been conducted concerning SPS spray process [1], fundamental understanding lags behind the applications, and there is limited research concerning droplet and particle behaviors. Marchand *et al.* [4] studied the influence of liquid injection angle, and results showed that the relative velocity between liquid drop size, drop flow and flow field and the surface tension of liquid drop have significant influence on the incident and fracture of the liquid. Huang *et al.* [5] adopted a stochastic approach to investigate the particle behavior with two-fluid parcels, and found that the existence of large-scale eddies, variable property, Knudsen effect and mass-transfer cooling effect would affect the particle motion and heating history. Fazilleau [6] found that the fragmentation and vaporization of suspension or droplets occur about 10 to 15mm downstream of the injection location, and solvent vaporization cools down the plasma jet. Ozturk and Cetegen [7] presented a physical model to analyze the droplet evaporation and particle behavior during plasma spraying. Jabbari *et al.* [8] used FLUENT software to simulate the liquid fragmentation without considering the droplet evaporation. Although some particular aspects of droplet and particle behavior have been studied, there is still a lack of comprehensive model that connects the suspension droplets to the nanoparticles. In addition, agglomerate sintering always occurs in SPS due to insufficient atomization and might degrade the nano-structured coating quality. Such effect is important but not well considered in these studies.

In this paper, a comprehensive three-dimensional model is presented to examine the suspension droplets and spray evolution after liquid atomization. Two sub-models are used. First, the primary breakup of annular liquid sheath is calculated by a one-dimensional model based on Lund [9]. Second, sub-model is the Eulerian/Lagrangian modeling of the plasma jet and injected droplets with suspended nanoparticles. The first sub-model provides the proper initial and boundary conditions for the second sub-model. Then, each atomized droplet motion and heating can be tracked in the three-dimensional geometry, considering the nanoparticles release and melting. After validation with published experimental data, this model is used to predict the droplet and particle evolution under different operating conditions. The influence of operating conditions on the nanoparticle release and melting are discussed.

## 2. Mathematic Model

As experiments observed, the flow structure at the near nozzle exit and the downstream jet, primary breakup and secondary breakup occur in different stages to fragmentize the liquid to droplets. In the following Section 2.1, three steps will be shown on how to calculate the primary breakup. After that, Section 2.2 will describe how the droplets experience further breakup in the high-velocity plasma jet, as well as other physical particle phenomena of acceleration, heating, melting and evaporation.

### 2.1. Modeling Liquid Primary Breakup to Estimate Droplet Mean Size

**Figure 1:**
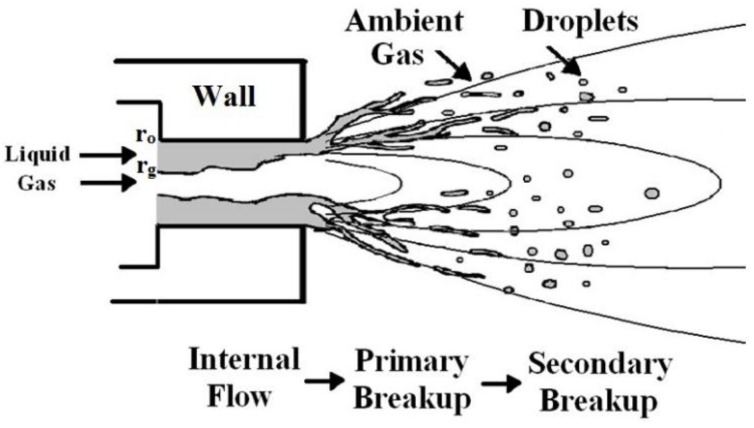
The schematic of liquid atomization.

Liquid primary breakup arises at the nozzle exit. There are three steps to fragmentize the continuous liquid into individual droplets. Firstly, the annular liquid sheath breaks up into a number of cylindrical ligaments, as indicated in Figure 1. Secondly, the ligaments breakup to ligament fragments. Finally, the ligament fragments stabilize to drops. To model droplet primary breakup, a one-dimensional primary breakup model, based on the one of Lund *et al.* [9], is described in this study and used to estimate the droplet mean size from the atomizing gas and liquid mass flow rates, liquid physical properties, and atomizer exit geometry. It is assumed that the annular liquid sheath breaks up to several cylindrical ligaments of almost same diameter as the thickness of the annular sheath. The ligaments further break up to ligament fragments at the wavelength of the most rapidly growing wave. Each fragment is assumed to form one droplet.

**Estimation of annular liquid sheath thickness:** To estimate the thickness of annular liquid sheath, a simple model is adopted. It is assumed that the annular two-phase flow within the discharge orifice is one-dimensional, in viscous and isothermal, with compressible ideal gas and small interface velocity slip ratio. The velocity of the gas flow, *v_g_*, satisfies the momentum equation as:

(1) $$\frac{dp}{\rho_{g}}+v_{g}dv_{g}=0$$

Using the state of relation, the integration of above equation gives out:

(2) $$RT\ln\left(\rho_{g}RT\right)+\frac{1}{2}{\left(\frac{{\dot{m}}_{l}ALR}{\rho_{g}\pi \text{​}r_{g}^{2}}\right)}^{2}=cosnt$$

where *r_g_* is the radius of gas flow, ρ*_g_*, the gas density, ${\dot{m}}_{l}$ is the mass flow rate of liquid and *ALR* is the air–liquid ratio by mass. The radius of gas flow can be written in terms of orifice radius, *r_o_*, using the definition of void fraction α as: $r_{g}=\sqrt{\alpha }r_{o}$. According to Ishii [10], the interface velocity slip ratio “*sr*” under different flow rate can be expressed as:

(3) $$sr=\sqrt{\frac{\rho_{l}}{\rho_{g}}\;\frac{\sqrt{\alpha }}{1+C\left(1-\alpha \right)}}$$

where *C* is the experimental coefficient. The interface velocity slip ratio and void fraction also has the relationship of:

(4) $$1+\frac{\rho_{g}sr}{\rho_{l}ALR}=\frac{1}{\alpha }$$

By solving Equations (2)–(4), ρ*_g_*, α and *sr* can be calculated for different operating conditions. The thickness of annular liquid sheath is then calculated as: $\delta =r_{o}-r_{g}$, which is also the diameter of the typical cylindrical ligament.

**Droplet mean size from primary breakup:** After obtaining the thickness of annular liquid sheath, we need to compute the length of a typical ligament fragment, *i.e.*, the wavelength λ at which the disturbance grows most rapidly. Among the linear instability analysis theories, the one given by Weber is mostly widely used to determine λ [11]:

(5) $$\lambda =\sqrt{2}\pi \;\delta \;\sqrt{1+\frac{3\mu_{l}}{\sqrt{\rho_{l}\sigma_{l}\delta }}}$$

However, this equation does not consider movement of the sheet and is approximately correct only in the case of long wave disturbances. A more accurate solution provided by Senecal *et al.* [12] is utilized, in which the growth rate of surface disturbances for the sinusoidal mode is given by:

(6) $$\begin{array}{l} \omega =-\frac{2\mu_{l}k^{2}\tanh\left(kh\right)}{\tanh\left(kh\right)+Q}\\ \;+\frac{\sqrt{4\mu_{l}^{2}k^{4}\tanh^{2}\left(kh\right)-Q^{2}V^{2}k^{2}-\left[\tanh\left(kh\right)+Q\right]\;\left(-QV^{2}k^{2}+\sigma_{l}k^{3}/\rho_{l}\right)}}{\tanh\left(kh\right)+Q} \end{array}$$

where *k* is the wave number given by *k =* 2π/λ, *h* is the half-thickness of sheath *h =* δ*/*2, *Q* is the gas/liquid density ratio, *V* is the liquid sheath velocity, and μ*_l_*, ρ*_l_*, and σ*_l_* represent the liquid viscosity, density, and liquid surface tension, respectively. Here, we use the relative velocity difference of the gas and the liquid at the nozzle exit. The breakup wavelength λ is determined by the wave number *k* where the maximum growth rate ω occurs in the curve calculated by Equation (6). Assuming that each fragment stabilizes to one droplet, the Sauter Mean diameter of drop size can then be calculated from the conservation of mass:

(7) $$\text{SMD}={\left[\frac{3}{2}\;\delta^{3}\lambda \right]}^{1/3}$$

Using this model, droplets mean diameter after primary breakup can be calculated and the initial droplet diameter is calculated based on this SMD diameter with a Rosin–Rammler distribution.

### 2.2. Eulerian/Lagrangian Model of Droplets or Particles in Plasma Jet

**Figure 2:**
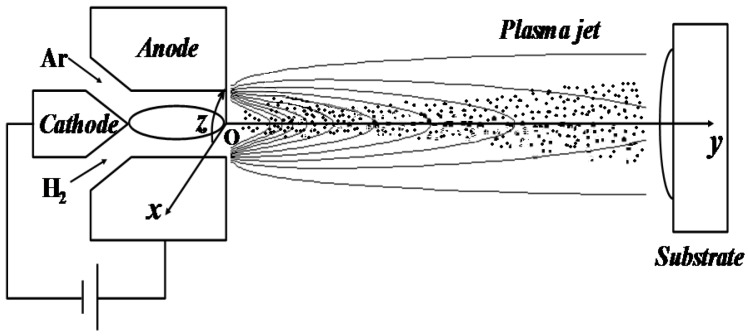
The schematic of suspension plasma spray with axially injected droplets or particles.

The droplets with imbedded solid particles experienced accelerating and heating in the plasma jet, and a code named LAVA-P-3D [13] was developed to simulate these processes as shown in Figure 2. The plasma gas flow fields were obtained by solving the Navier-Stokes (N-S) equations in Eulerian method, and tracking the particles as Lagrangian entities. The N-S equations of the plasma jet were established by assuming that the plasma jets were continuum, multi-component, compressible and chemically reactive, with temperature-dependent transport properties in the local thermodynamic equilibrium. The turbulence was simulated using *k-*ε model. Details of the plasma jet simulation could be found in reference [13].

For the droplets injected into the plasma jet, their evolution include droplet secondary breakup, solvent evaporation, particle acceleration, heating and melting. These behaviors were simulated during their flight. In plasma flow field, the volume fraction of particles is less than 10^−4^, so the collisions between particles were neglected [13].

**Figure 3:**
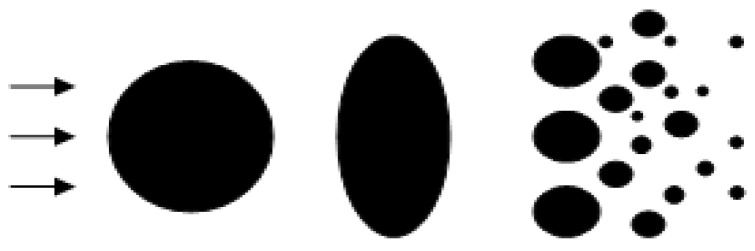
Schematic of droplet secondary breakup.

**Droplet secondary breakup modeling:** When the droplets with initial diameter estimated from Equation (7) were injected into the plasma jet, secondary atomization would continually breakup the droplets, especially near the orifice with strong aerodynamic interaction. To model the droplet secondary breakup, a cascade atomization and droplet breakup (CAB) model as shown in Figure 3 [14] has been utilized, which is determined by the gas aerodynamic force, the liquid viscosity, and the surface tension force.

In this model, the droplet distortion is described by the deformation parameter, *y* = 2*x*/*r*, where *x* denotes the radial cross-section change from its equilibrium position and *r* is the drop radius. The deformation parameter is calculated by [15]:

(8) $$\ddot{y}+\frac{5\mu_{l}}{\rho_{l}a^{2}}\dot{y}+\frac{8\sigma }{\rho_{l}a^{3}}y=\frac{2\rho_{g}{\left|U\right|}^{2}}{3\rho_{l}a^{2}}$$

where ρ denotes the density, μ the viscosity, σ the surface tension, *U* the relative drop-gas velocity, and the subscripts *g* and *l* denote the gas or liquid properties, respectively. Drop breakup occurs when the normalized drop distortion, *y*(*t*), exceeds the critical value 1.

The creation of the product droplets is derived by using elements from population dynamics: for each breakup event it is assumed that the number of product droplets is proportional to the number of critical parent drops, where the proportionality constant depends on the drop breakup regime. From this, one can define the rate of droplet creation, which, in conjunction with the mass conservation principle between parent and product droplets, leads to the basic cascade breakup law:

(9) $$\frac{d}{dt}\bar{m}(t)=-3K_{bu}\bar{m}(t)$$

where *m*(*t*) denotes the mean mass of the product drop distribution, and the breakup frequency *K_bu_* depends on the drop breakup regimes. As suggested by Reitz [16], three breakup regimes are classified with respect to increasing gas Weber number, bag breakup, stripping breakup regime, and catastrophic breakup regime. However, in this study, gas Weber number is mostly lower than 80, which falls into bag breakup regime, as shown in Figure 3. Here, Weber number provides the importance of inertia compared to surface tension. Breakup frequency $K_{bu}=0.05\omega$ as suggested by O’Rourke and Amsden [15] is used in this study, and the drop oscillation frequency ω is given by

(10) $$\omega^{2}=\frac{8\sigma }{\rho_{l}a^{3}}-\frac{25\mu_{l}^{2}}{4\rho_{l}^{2}a^{4}}$$

Note that, except for the mean mass *m*(*t*), the actual size distribution of the product droplet has not been specified yet. For the model implementation in this study, a uniform product drop size distribution has been assumed, by which Equation (9) becomes:

(11) $$\frac{r}{a}=e^{-K_{bu}t_{bu}}$$

where *a* and *r* are the radii of the parent and product drops, respectively, and *t_bu_* is the breakup time, *i.e.*, the time until the normalized deformation *y*(*t*) in the solution of Equation (8) exceeds the value of 1.

**Figure 4:**
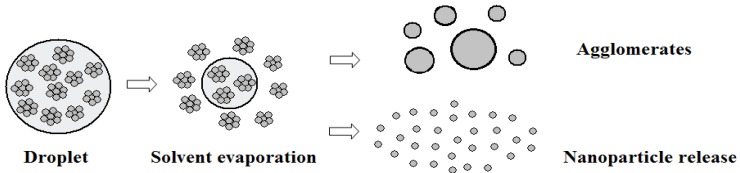
Schematic of droplet injection, solvent evaporation and nanoparticle release. Reproduced with permission from [17]. Copyright Courtesy of Delbos, 2006.

**Solvent evaporation of droplets:** The suspension droplets would evolve to agglomerates and nanoparticles due to solvent evaporation. As shown in Figure 4, the nano-sized solid particles were initially suspended in the micro-sized droplets, with small amount of particle aggregation, and uniform distribution. The aerodynamic interaction between the plasma gas and the droplet would further breakup the droplets into smaller pieces. At the same time, the hot plasma gas would also heat up and vaporize the solvent inside droplets. Once the solvent in droplets was totally vaporized out, the gas would blow away the solids to form individual nanoparticles. Otherwise, if the gas velocity is not large enough, the remaining solids would be sintered by the hot gas and the micro-size agglomerates would form thereafter. In this study, the nanoparticle size is larger than 1 nm but less than 1 μm, and the agglomerate is larger than or equal to 1 μm. These agglomerates or nanoparticles are simulated as new Lagrangian entities with their parent particle’s position, velocity and temperature.

The droplet temperature can be expressed by:

(12) $$\begin{array}{l} T_{d}=T_{d,0}+\frac{Q_{d}}{m_{d}c_{p,d}}t\;if\;T_{d}<T_{m,d}\\ T_{d}=T_{m,d}\;if\;m_{d}c_{p,d}\left(T_{m,d}-T_{d,0}\right)\leq Q_{d}\leq m_{d}c_{p,d}\left(T_{m,d}-T_{d,0}\right)+m_{d}\alpha_{sl}L_{v,sl} \end{array}$$

where *T_d,_*_0_, *Q_d_*, *m_d_* and *c_p,d_* are the initial droplet temperature, heat gain, mass, and the specific heat of the droplet, respectively. *Q_d_* can be calculated by *Q_d_ = Q_conv_ − Q_rad_*, where *Q_conv_* represents the convection heat, and *Q_rad_* is the radiation heat loss of particles. *c_p,d_* is calculated based on the average of the mass fraction of solid particle and solvent as: $c_{p,d}=c_{p,p}\left(1-\alpha_{sl}\right)+c_{p,sl}\alpha_{sl}$.

When the droplet solvent is totally vaporized, the solid particles contained in the droplet will be released into the plasma jet. They might be released as micrometer-sized agglomerates containing many nano-sized particles or individual nano-sized particles due to the evaporation of solvent and the aerodynamic forces of the plasma gas [18]. These individual micro- or nano-sized particles are treated as new Lagrangian entities with the current parameters of their mother particle, including position, droplet velocity, and temperature.

**Particle acceleration and tracking model:** For particles in plasma jets, the forces imparted on the particles are mainly the drag force, Saffman lift force and Brownian force. For particles smaller than 100 μm, the drag force is prominent. For the particles near the jet edge and the substrate, where the flow shear stress is large, the Saffman lift force is significant. While for the sub-micron or nanoparticles, Brownian force is important. By accounting for these three forces, the particles acceleration rate could be expressed as,

(13) F→=F→drag+F→Saffman+F→Brownian=mpdV→pdtF→drag=mp38ρ¯ρpCDrp|V→g−V→p|(V→g−V→p)F→Saffman=mp2Kc(μρg)0.5dijρgρpdp(dlkdkl)0.25(V→g−V→p)F→Brownian=mpG0πS0Δt

where *V_g_* is the gas velocity within the turbulent fluctuation calculated from the gas turbulence model. During the particle tracking procedure, the turbulent dispersion of particles is calculated by integrating the trajectory equations for individual particles, using the instantaneous fluid velocity along the particle path. *C_D_* is the drag force coefficient expressed by [19],

(14) CD=(24Rep+61+Rep+0.4)fprop−0.45fKn0.45

in which the particle Reynolds number is a dimensionless number providing the importance of inertia compared to viscous force and defined as:

(15) Rep=2ρgrp|V→g−V→p|/μ

*f_prop_* represents the effects of variable plasma properties in the boundary layer surrounding the particle, and can be expressed as [20] $f_{prop}=\rho_{c}\mu_{c}/\rho_{w}\mu_{w}$.$f_{Kn}$ is the factor representing Knudsen effect, which can be expressed by:

(16) $$f_{Kn}={\left[1+\left(\frac{2-a}{a}\right)\left(\frac{\gamma_{w}}{1+\gamma_{w}}\right)\frac{4}{\mathrm{Pr}}Kn\right]}^{-1}$$

where *a* is the thermal accommodation factor, usually with the value of 0.8 [21]; Pr is dimensionless Prandtl number which provides the importance of momentum transfer compared to heat transfer; *Kn* is the Knudsen number for the importance of small scale effect and defined by the effective mean free pathλ and the droplet diameter, $Kn=\lambda /d_{p}$, where $\lambda =2\mu /\left(\rho_{g}v_{w}\right)$; and $v_{w}$ is the mean molecular speed that is dependent on the gas temperature near the particle surface *T_w_*, as well as the average molecular weight *W* of the gas mixture, and can be given as: $v_{w}={\left(8RT_{w}/\pi W\right)}^{1/2}$. For nanoparticles, $f_{Kn}$ is in the range 0.005 to 0.1. For the agglomerates and micro-sized particles, $f_{Kn}$ changes from 0.994 to 0.996 [22].

*K_c_* = 2.594 is the constant in the Saffman lift force [23], *d_ij_* is the deformation tensor. In the expression of Brownian force, *G_0_* is a random number between −1 to 1, which is subjected to Gauss distribution. *S_0_* is the spectral intensity, which can be expressed as $S_{0}=\left(216{\mu \sigma }_{B}T_{g}\right)/\left(32\pi^{2}r_{p}^{5}\rho_{p}^{2}C_{c}\right)$, Boltzman constant σ*_B_* = 1.38 × 10^−23^ J/K.

Equation (13) is used to depict the particle velocity and trajectory. The local gas conditions around the particle are employed to calculate the particle heating.

**Heating and melting of particles:** A one-dimensional model was adopted for the particle heating and melting, in which the spherical shape of the particle was assumed. The internal convection within the molten part of the particle was not considered. The temperature distribution inside the particle was described as follows:

(17) $$\rho_{p}C_{p}\frac{\partial T_{p}}{\partial r}=\frac{1}{r^{2}}\frac{\partial }{\partial r}\left(k_{p}r^{2}\frac{\partial T_{p}}{\partial r}\right)$$

Zero temperature gradient was assumed in the particle center. The particle surface was subjected to energy conservation law, as [24]:

(18) $${\frac{\partial T_{p}}{\partial r}|}_{r=0}=0,and\;{4\pi r_{p}^{2}\left(k_{p}\frac{\partial T_{p}}{\partial r}\right)|}_{r=r_{p}}={\dot{Q}}_{conv}-{\dot{Q}}_{vap}-{\dot{Q}}_{rad}$$

where the convection, evaporation latent and radiation heat rates (${\dot{Q}}_{conv}$, ${\dot{Q}}_{vap}$ and ${\dot{Q}}_{rad}$) are expressed as $4\pi \;r_{p}^{2}\;h_{f}\left(T_{f}-T_{s}\right)$, ${\dot{m}}_{v}L_{v}$ and $4\;\pi \;r_{p}^{2}\;\varepsilon_{p}\;\sigma_{s}\;\left(T_{s}^{4}-T_{\infty }^{4}\right)$, respectively. The film temperature, *T_f_* was defined as (*T_s_ + T_g_*)/2, which is introduced to deal with the steep temperature gradient in the boundary layer around the particle. Only the radiation between the particle surface and the environment was considered in the case of optically thin plasma gas. The heat transfer coefficient, *h_f_*, can be calculated from [25]:

(19) Nu=2hfrpkf=(2.0+0.6Rep1/2Pr1/3)fpropfKnfv

where *f_v_* accounts for the effect of mass transfer due to evaporation, which can be found in reference [22]. Additional constraints of energy balance between the heat conduction and latent heat at the melting interface *r_m_* was also considered:

(20) $${{\left(k_{p}\frac{\partial T_{p}}{\partial r}\right)|}_{r=r_{m}^{-}}-\left(k_{p}\frac{\partial T_{p}}{\partial r}\right)|}_{r=r_{m}^{+}}=L_{m}\rho_{p}\frac{dr_{m}}{dt\;}$$

## 3. Experimental and Numerical Setup

The present study was conducted for a direct-current suspension plasma spray system with axial injection of feedstock as shown in Figure 2. The ZrO_2_ nanoparticles were suspended in the alcohol solvent, as shown in Figure 4, with solid weight content of 10%. Gas mixtures of argon and hydrogen were ionized in plasma gun to form high temperature and high velocity plasma jet. The droplets and particles were accelerated and heated in the plasma gas. At last, the melting nanoparticles formed coatings on the substrate.

For the plasma gas, the Ar and H_2_ gas flow rates are 68 and 12 liters per minute, respectively. The electric power input of the plasma torch gun is 30 kW. The plasma flow field was solved using a three-dimensional cylindrical coordinate system. The radial distance was 6 cm with 57 grid points, the axial distance was 15 cm with 66 grid points, and the circular direction was 2π with 32 grid points. At the axis of the gas field, the symmetrical condition is applied. At the nozzle exit, the velocity and temperature could be expressed by the empirical formulae [26], *v*(*r*) = *V_cl_*[1 − (*r*/*R_i_*)^1.2^], and *T*(*r*) = (*T_cl_* − *T_w_*)[1 − (*r*/*R_i_*)^6^] + *T_w_*, respectively. *V_cl_* and *T_cl_* are the velocity and temperature on the nozzle exit center line, respectively, which are calculated from the total amount of momentum and thermal energy transferred to the plasma jet. *T_w_* is the wall temperature with the initial value of 300 K, the velocity at the wall boundary is 0. The downstream of the jets flow is open.

The droplets and particles were being tracked in the plasma flow field. The droplets had an initial temperature of 300K, and initial velocity from 50 to 500 m/s. The particle temperature distribution and melting interface were calculated using 50 grid points in spherical coordinates for each solid particle.

## 4. Validation of Model Predictions

**Figure 5:**
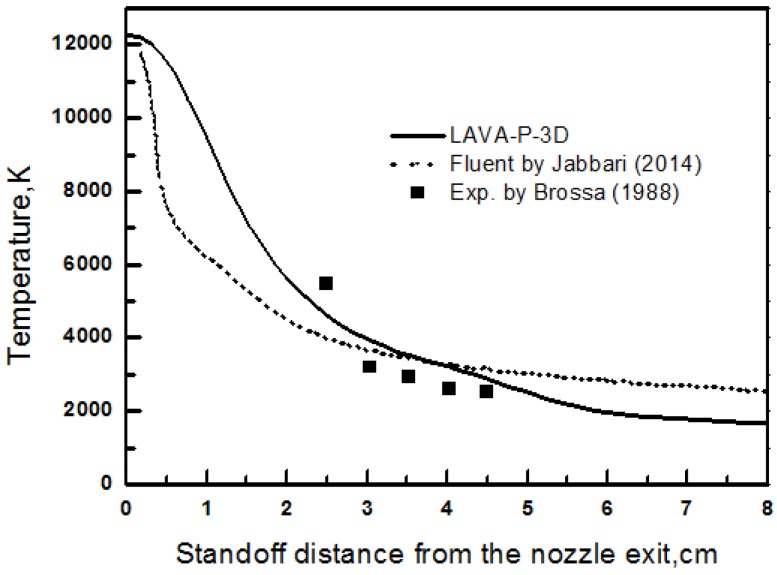
Comparison of gas temperature with published numerical (Reproduced with permission from [8]. Copyright Courtesy of Jabbari, 2014) and experimental data (Reproduced with permission from [27]. Copyright Courtesy of Brossa, 1988).

In order to verify the numerical code LAVA-P-3D for plasma spray process, the centerline gas temperature profile were calculated and compared with published numerical results [8] and experimental data [27] in Figure 5. A good agreement between the simulation results and experimental data has been obtained. The deviation between experiment and our modeling result is within 10%, which shows a better accuracy comparing to the FLUENT simulation.

## 5. Discussion

### 5.1. Flow Field of the Plasma Jet

**Figure 6:**
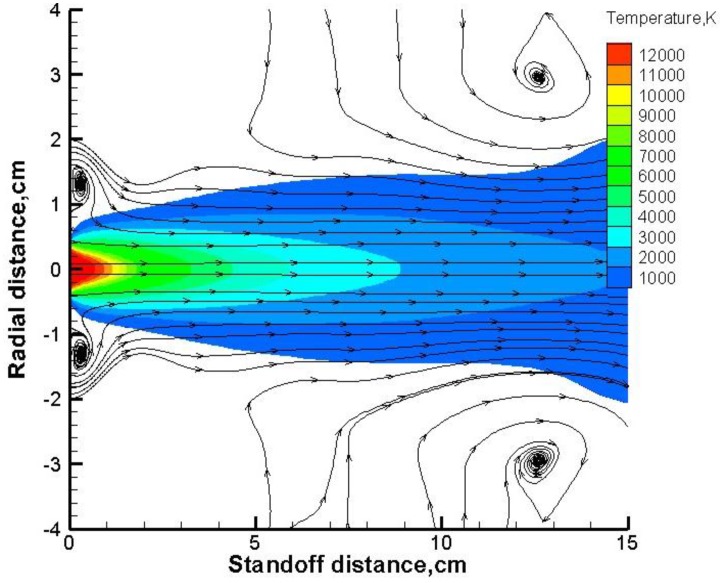
Gas temperature contours in suspension plasma spray.

As shown in Figure 6, the hottest plasma zone extends from the torch exit to 1 cm downstream, with temperature as high as 10,000 K. Beyond this core region, the gas temperature cooled down continuously along the spray distance. The gas formed two vortexes near the nozzle exit and another two vortexes in the far field. The vortexes near the exit were formed because of the torch walls besides the exit and the steep shear layer at the outer edge of the jet induced those two vortexes in the far field. We have also noted that vortex location in the far field was not fixed, depending on the specific flow field environment.

### 5.2. Effects of Droplet Diameter on Nanoparticle Release

**Figure 7:**
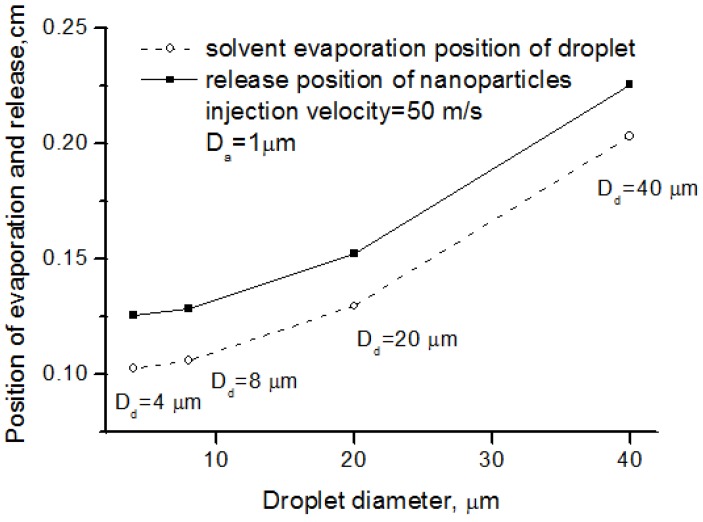
Solvent evaporation position and nanoparticle release position for different droplet diameter.

The sizes of droplet and agglomerate controlled the solvent evaporation and nanoparticle release. Proper atomization parameters contributed to better nanoparticle release. Figure 7 depicts the nanoparticle release position, as well as the solvent evaporation position, from different size of suspension droplets. The solvent evaporation position and nanoparticle release position is the location where solvent evaporation and nanoparticle release take place, respectively, as illustrated in Figure 4. The larger the droplet diameter was, the latter the release position was. This means that the nanoparticle processing in SPS preferred the good atomization and small droplet size.

**Figure 8:**
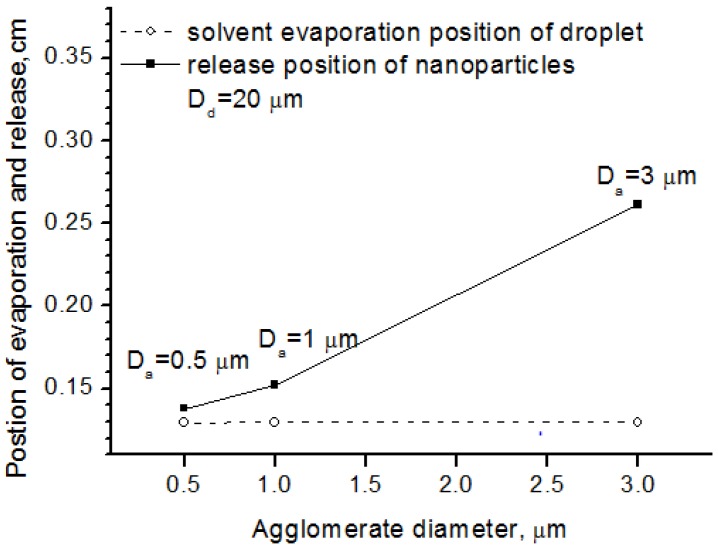
Solvent evaporation position and release position for different agglomerate diameter.

Figure 8 shows the effects of the agglomerate size on nanoparticle release position, where the droplet diameter was the same. From the picture, we can see that large agglomerate size lengthened the standoff distance between the nanoparticle release and the solvent evaporation, because large agglomerates need more energy to blow the nanoparticles off into the flow field. It should be mentioned that the agglomerate here is not produced from sintering as discussion in the following Section 5.4 and Section 5.5. Instead, they are induced from the aggregation effect when preparing the suspension. Thus, it could also be concluded that the nanoparticle processing in SPS preferred good disintegration between the solid and the liquid for suspension preparation.

### 5.3. Effects of Atomization Injection Parameters on Nanoparticle Release

The injection angle and injection velocity are key atomization parameters that affect the nanoparticle release. Injection angle, or cone angle, means the angle between the two outer edges of the spray. Zero cone angle indicates that the spray droplets are injected in the centerline. In experiments, cone angle is larger than zero and here we used cone angles of 10 and 20 degrees. We also defined the nanoparticle release percentage as the ratio of the released nanoparticle mass over the total solid mass.

**Figure 9:**
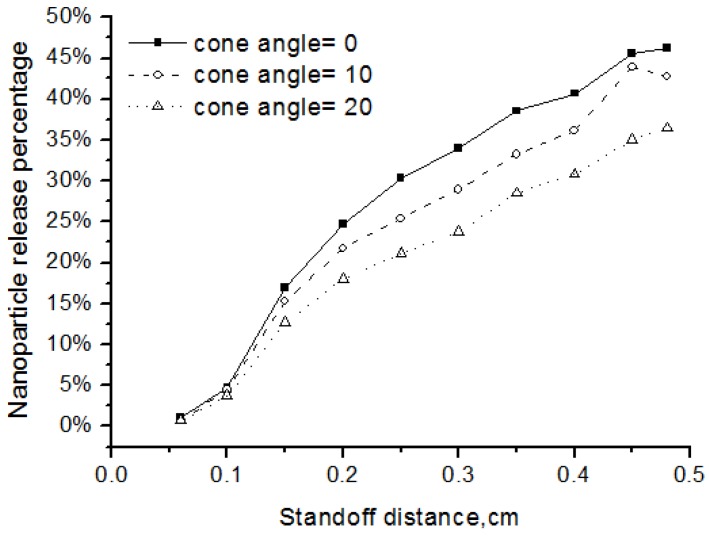
Effects of injection angle on the nanoparticle release percentage.

**Figure 10:**
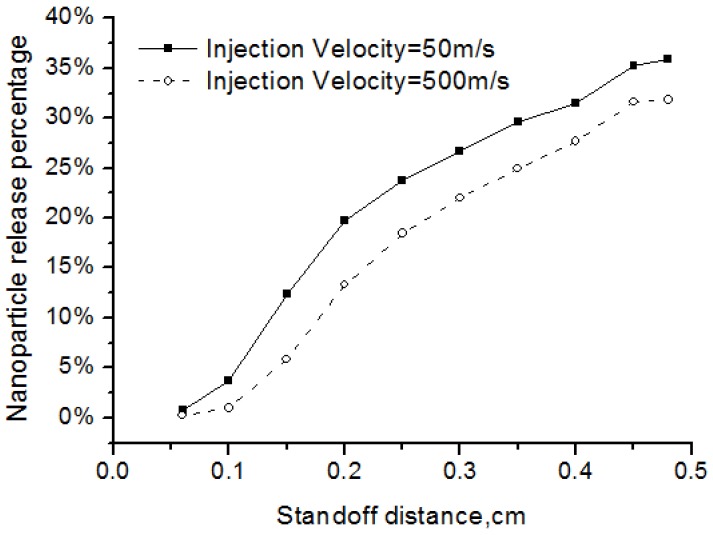
Effects of injection velocity on the nanoparticle release percentage.

As shown in Figure 9, when the injection angle became larger, fewer nanoparticles are released into the flow field. The reason is more particles would be distributed at the fringe of the flow field and could not be well heated up if the injection angle is larger. The particle injection velocity also affected the release percentage of the nanoparticles. Two cases with different initial particle injection velocity of 50 m/s and 500m/s are simulated. Results in Figure 10 showed that, when the injection velocity increasing, the release percentage of nanoparticles became smaller. This is because the high-velocity particles have short flight and heating time in the high-temperature flow field. In addition, the results showed that the nanoparticle release occurred early before the standoff distance of 0.4 cm, since the solvent evaporation point is pretty low at about 350 K while the plasma gas in this region is much higher.

### 5.4. Velocity, Temperature and Melting of Nanoparticles and Agglomerates

As shown in Figure 4, many suspension droplets would be fragmentized into small pieces by aerodynamics force, which would be released into nano-sized particles after solvent evaporation. However, some of the suspension droplets might not experience enough aerodynamics force to further breakup the droplets (see Figure 3), which would be sintered into a micro-sized solid agglomerate after solvent evaporation. Those released nano-sized particles and sintered micro-sized agglomerates have different heating and accelerating processes.

**Figure 11:**
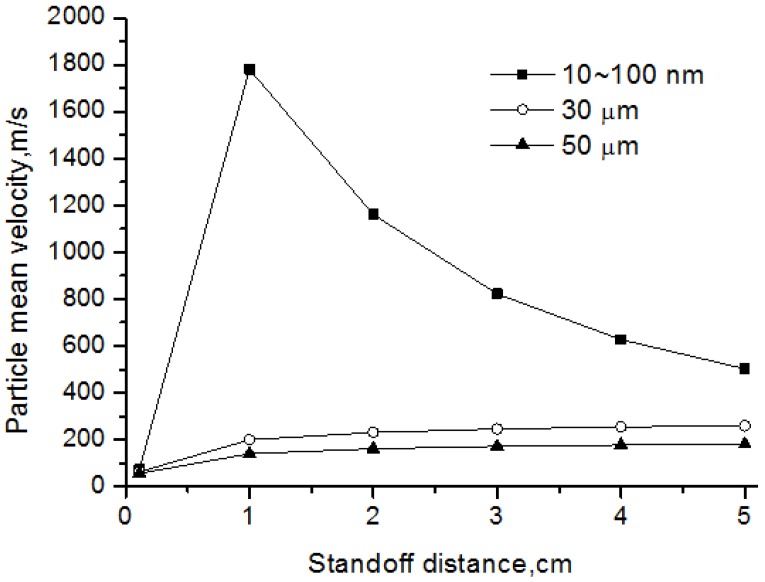
Velocity of nanoparticles and agglomerates.

Figure 11 represents the particle velocity for nanoparticle and agglomerate with different diameter. In this study, the released nanoparticles have diameter ranging from 10 to 100 nm. Results showed that the small inertia of nanoparticles improved the acceleration process, especially in the hottest plasma zone within 1 cm from the exit where the plasma velocity is also very high. However, the nanoparticles decelerated rapidly behind this zone, for the reason of slowing plasma gas. This means that the nanoparticles show good tracking characteristics, and their impact velocity before coating mainly depends on the local gas velocity. For the micro-sized agglomerates, their acceleration processes were much less than that of nanoparticles due to their large inertia. These micron particles usually keep accelerating during their flight. Therefore, their impact velocity mainly depends on the speeding rate, and the relatively small micro-sized agglomerates have high impact velocity.

**Figure 12:**
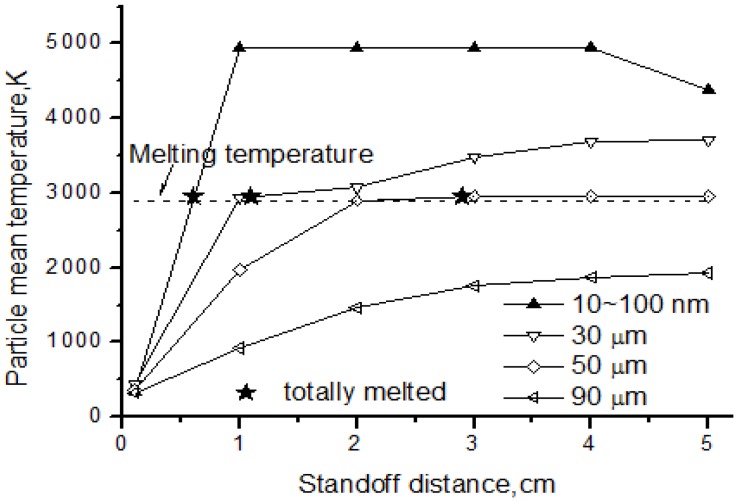
Temperature of nanoparticles and agglomerates.

The particle temperature and melting status are shown in Figure 12 for nanoparticles and agglomerates with different diameter. From the picture, we can see that the nanoparticles could be easily melted when reaching melting temperature at the standoff distance 0.6 cm. After being totally melted, the nanoparticles continued heating, and the temperature could reach the solid evaporation point as high as 5000 K. For the agglomerates, the heating rate was much less than that of the nanoparticles. The larger the particle was, the lower the temperature reached. For this reason, some agglomerates could be melted while others could not. Results showed that the 30 μm agglomerate was totally melted at the distance of 1.1 cm and the 50 μm agglomerate was melted at 2.9 cm, but the 90 μm agglomerate never reached the melting temperature. The larger the particles were, the more heat needed to absorb from the flow field to reach melting status. Therefore, the melting position was delayed more to the rear of flow field if increasing the particle diameter. This also indicated that the solvent evaporation was not a key factor for agglomerate temperature and melting, since evaporation had been done early before standoff distance about 0.4 cm.

### 5.5. Critical Agglomerate and Droplet Size

Particle diameter was important to their melting status before coating, especially for agglomerates. We define the melting percentage for nanoparticles and agglomerates, as the ratio of the melted mass over the initial solid mass during their flight.

Table 1 lists the maximum of melting percentage for nanoparticles and agglomerates with different size, as well as the standoff position where this maximal melting finished. Results showed that all the nanoparticles could be easily melted once they were released and heated up to melting temperature. For micro-sized agglomerates, things were different in two aspects. Firstly, their melting percentage decreased continuously with the particle size increased. Secondly, their flight distance before being mostly melted was much longer comparing to the nanoparticles. One reason is that the heat transfer efficiency of agglomerates is lower than that of the nanoparticles. Another reason is that the agglomerates have large latent heat at the melting interface and heat conduction needs a long flight time to balance it. Poorly melted particles would reduce the coating quality and their existence should be avoided. For this reason, the agglomerate size should be limited to a critical number, as well as the droplet size.

Our calculation showed the critical agglomerate diameter being totally melted is 50 μm for current SPS conditions. The agglomerates smaller than this size could be totally melted and those larger than it could only be partially melted or not melted at all. Our previous results showed that the solvent evaporation was not a key factor for agglomerate temperature and melting. Thus, we could assume that a suspension droplet with diameter of *D_d,crit_* and solid weight content of *wt*, experienced no secondary atomization and was sintered to one agglomerate with diameter of *D_a,crit_*, *i.e.*, 50 μm in this case. Then, the critical suspension droplet size, *D_d,crit_* could be estimated as:

(21) $$D_{d,crit}=D_{a,crit}{\left(wt\right)}^{-1/3}$$

For the typical weight content of 10%–25%, the critical suspension droplet size could be calculated as 80–100 μm for current suspension plasma spray. Lower weight content had larger critical droplet size. Such estimation agrees fairly well with what Fauchais reported in 2008, that the droplet diameter should be limited below 90 μm for better particle melting [28].

## 6. Conclusions

In our present work, a comprehensive model has been developed to simulate the evolution of suspension droplet, nano-sized particles, as well as micro-sized agglomerates in the high-temperature plasma jet. This model simultaneously studied the dynamic, thermal and phase change behaviors of nano-sized or micro-sized particles. To validate this model, we compared our numerical results with other group’s experiments as well as simulation work, proving its accuracy with deviation less than 10%. Using this model, effects of atomization parameters on the nanoparticle processing have been thoroughly studied. Proper conditions are concluded to ensure nanoparticle release and agglomerate melting during suspension plasma spray.

Firstly, the effects of atomization parameters on the nanoparticle release, including droplet diameter, injection angle and velocity, were investigated. The study reveals that the nanoparticle processing in SPS preferred small droplets with better atomization and less aggregation from suspension preparation. The injection angle and velocity influenced the nanoparticle release percentage. Small angle and low initial velocity might have more nanoparticles released.

Besides, the melting percentage of nanoparticles and agglomerates were studied, and the critical droplet diameter to ensure solid melting was drawn. Results showed that for better solid melting, the suspension droplet size should be limited to a critical droplet diameter, which was inversely proportional to the cubic root of weight content of the given critical agglomerate diameter of being totally melted. Of course, this critical agglomerate diameter would depend on the plasma condition, the particle material and the substrate distance, which could be further studied in the future.

## Figures and Tables

**Table 1:** Melting percentage and position for nanoparticles and agglomerates.

Particle Diameter	Maximum of Melting Percentage	Mostly Melted Position
10–100 nm	100%	6 mm
30 μm	100%	11 mm
40 μm	100%	15 mm
50 μm	100%	29 mm
60 μm	93.26%	38 mm
70 μm	76.40%	62 mm
80 μm	2.25%	74 mm
90 μm	0	None

## Nomemclature

*C_D_*	drag coefficient
*C_p_*	specific heat, J·kg^−1^·K^−1^
*D_d_*	droplet diameter, μm
*D_a_*	agglomerate diameter, μm
*d_ij_*	deformation tensor
*d_p_*	particle diameter, m
*f_Kn_*	factor ofKnudsen effect
*f_prop_*	factor to account property variation
*f_v_*	factor formass transfer
*G_0_*	zero-mean, unit-variance independent Gauss random number
*h*	heat transfer coefficient, W·m^−2^·K^−1^ or the half-thickness of sheath
*k*	thermal conductivity, W·m^−1^·K^−1^ or wave number
*K*_c_	constant coefficient in Saffman lift force
*Kn*	Knudsen number
*L_m_*	latent heat of fusion, J·kg^−1^
*L_v_*	latent heat of vaporization, J·kg^−1^
*m_p_*	particle mass, kg
${\dot{m}}_{v}$	vaporization rate, kg·s^−1^
*Nu*	Nusselt number
p	pressure, Pa
Pr	Prandtl number, *Pr =* μ*C_p_k*^−1^
*Q*	heat flux, W·m^−2^ or the gas/liquid density ratio
*R*	gas constant, J·mol^−1^·K^−1^
*r*	radial coordinate for the particle, m
$r_{m}^{-}$	inner radius of melting interface, m
$r_{m}^{+}$	outer radius of melting interface, m
*r_m_*	position of particle melting interface, m
*r_p_*	particle radius, m
*S_0_*	spectral intensity
SMD	Sauter mean diameter, μm
*T*	temperature, K
*t*	time, s
*T_∞_*	temperature outside boundary layer, K
*T_d,0_*	droplet initial temperature, K
*T_m,d_*	droplet melting point, K
*T_s_*	particle surface temperature, K
*T_w_*	gas temperature near particle surface, K
*v_g_*	gas velocity, m·s^−1^
*V*	velocity, m·s^−1^
*v_w_*	mean molecular speed, m·s^−1^
*W*	molecular weight of thegas mixture, kg·mol^−1^
*Weber*	Weber number, dimensionless
*wt*	solid weight content in suspension, dimensionless
*y*	deformation parameter, dimensionless
σ_*s*_	Stefan-Boltzmann constant

## Greek symbols

ρ	density, kg·m^−3^
μ	viscosity, kg·s^−1^·m^−1^
γ_w_	specific heat ratio of gas, dimensionless
α	weight fraction or void fraction
ε	surface emissivity coefficient
δ	the thickness of annular liquid sheath
σ	surface tension
ω	the growth rate of surface disturbances

## Subscript

*d*	suspension droplet embedded with nanoparticles
*f*	film around the particle
*g*	plasma gas
*p*	solid nanoparticles or agglomerates
*sl*	solvent
